# Pancreatic stone protein inhibits pyroptosis of pancreatic acinar cells in sepsis-associated pancreatic injury

**DOI:** 10.3389/fmed.2025.1566728

**Published:** 2025-07-10

**Authors:** Pingping Liu, Zhenghui Xiao, Xiulan Lu, Xinping Zhang, Jiaotian Huang

**Affiliations:** Department of Emergency & Key Laboratory of Pediatric Emergency Medicine of Hunan Province, The Affiliated Children's Hospital of Xiangya School of Medicine, Central South University (Hunan Children's Hospital), Changsha, Hunan, China

**Keywords:** pancreatic stone protein, pyroptosis, sepsis, pancreatic injury, regenerating protein

## Abstract

**Introduction:**

Sepsis-associated pancreatic injury (SPI) is characterized by an increased incidence and significantly higher mortality rates. However, its underlying pathogenesis remains inadequately understood. As an acute-phase protein secreted by the pancreas, the role of pancreatic stone protein/regenerating protein (PSP/reg) in SPI remains unclear.

**Materials and methods:**

A total of 137 patients were consecutively admitted to the pediatric intensive care unit (PICU) of Hunan Children's Hospital with sepsis were included. A sepsis-associated pancreatic injury mice model was established using cecal ligation and puncture. Pancreatic injury was assessed using HE staining. Pyroptotic pancreatic cells were evaluated via Hoechst 33342/PI staining. The expression levels of caspase-1 in the pancreatic tissues and acinar cells were determined by immunohistochemistry and immunofluorescence. Protein levels of NLRP3, caspase-1 p20, and GSDMD-N were analyzed using Western blot. The concentrations of TNF-α, IL-6, IL-1β, and IL-18 were measured by ELISA.

**Results and discussion:**

Children in dead group exhibited higher circulating PSP/reg levels compared with children in survival group. The circulating PSP/reg level in the septic shock group was significantly higher than that in the sepsis group and the severe sepsis group. The circulating PSP/reg level in the severe elevation of pancreatic amylase group was significantly higher than that in the normal pancreatic amylase group and the mild elevation of pancreatic amylase group. Administration of PSP/reg significantly mitigated pancreatic injury, as evidenced by reduced histological scores and necrotic areas. The amylase activity, the serum levels of LDH, TNF-α, and IL-6 were remarkably downregulated in PSP/reg-treated mice compared to the SPI mice. PSP/reg administration significantly alleviated the LPS-induced pyroptosis. Pyroptosis activation-associated proteins, NLRP3, Caspase-1 p20 and GSDMD-N in pancreatic acinar cells were greatly elevated following LPS stimulation, which decreased with PSP/reg treatment.

**Conclusion:**

PSP/reg may exhibit protective effects by inhibiting pancreatic pyroptosis in sepsis-associated pancreatic injury model and LPS-stimulated pancreatic acinar cells.

## 1 Introduction

Sepsis remains a significant contributor to morbidity and mortality among children. The incidence of pediatric sepsis in intensive care units is exhibiting an increasing trend. Currently, there is no effective therapy available for sepsis, primarily due to an incomplete understanding of its underlying pathogenesis (1). The pathogenesis of sepsis appears to be complex, involving a range of factors including metabolic dysfunction, excessive oxidative stress, epigenetic reprogramming, intestinal barrier failure, immune dysregulation, neuroendocrine disorders, coagulation abnormalities and numerous other issues (2). Among the different forms of organ dysfunction associated with sepsis, pancreatic injury is associated with markedly higher mortality rates in comparison to dysfunctions affecting other organ systems (3). Our research group has also demonstrated that pancreatic injury serves as an independent risk factor for mortality (4). Therefore, revealing novel mechanisms that underlie acute pancreatic injury may facilitate the development of innovative therapeutic strategies.

Pancreatic stone protein/regenerating protein (PSP/reg) was first identified in pancreatic juice as lithostathine and regenerating protein 1 (Reg I) during the 1980s, and to a lesser extent, it expressed in the gastric mucosa and the kidney. PSP/reg, a low-molecular-weight protein with an approximate mass of 14 kDa, exhibits structural similarities to C-type lectin-like proteins that are associated with inflammatory responses (5). In cases of sepsis, the pancreas detects injury in remote organs and secretes PSP/reg without causing harm to pancreatic tissue, thereby designating PSP/reg as an acute-phase protein. Emerging evidence indicates that PSP/reg is emerging as a highly sensitive biomarker for the confirmation of sepsis and the prediction of mortality in critically ill pediatric patients. The integration of the PSP/reg biomarker into routine clinical practice has the potential to enhance the management of pediatric sepsis (6). Previous research has shown that PSP/reg was upregulated in rat pancreas after induction of acute pancreatitis, which suggested that induction of PSP/reg may be important for recovery from acute pancreatitis (7). In addition, As PSP/reg is a small secreted peptide, it holds promise as a biomarker of beta cell endoplasmic reticulum stress in the pathogenesis of diabetes (8). However, the role and molecular mechanism of PSP/reg in sepsis-associated pancreatic injury (SPI) remains poorly understood.

Pyroptosis is a form of programmed cell death that is characterized by cellular swelling and osmotic lysis, leading to the rupture of the cytoplasmic membrane and the subsequent release of immunostimulatory components. These components play a significant role in various pathological processes (9). Significant cellular responses to various stimuli involve the formation of inflammasomes, the maturation of inflammatory caspases, and the caspase-mediated cleavage of gasdermin. Inflammatory diseases, such as sepsis, are associated with uncontrolled pyroptosis (10). Pyroptosis is intricately associated with organ dysfunction induced by sepsis, and current strategies aimed at modulating pyroptosis primarily focus on targeting inflammasomes, key caspase enzymes, GSDMD proteins, and downstream inflammatory mediators (11). Therefore, investigating the role and molecular mechanisms of pyroptosis in sepsis-related organ dysfunction holds significant importance for the prevention and treatment of sepsis. It has been suggested that PSP/reg may have anti-apoptotic characteristics in acute pancreatitis (12). However, whether PSP/reg is related to pyroptosis in SPI remains unclear.

Based on the important role of pyroptosis and PSP/reg in sepsis, we investigated the role and regulatory function of PSP/reg on pyroptosis of pancreatic acinar cells to further confirm the precise function of PSP/reg involved in the pathogenesis of sepsis-associated pancreatic injury.

## 2 Materials and methods

### 2.1 Study population

From Jan 2022 to Jun 2023, a total of 137 patients were consecutively admitted to the pediatric intensive care unit (PICU) of Hunan Children's Hospital with sepsis were included. Some patients with acute pancreatitis, parotitis, diabetes mellitus and pancreatic primary tumor were excluded. The definition of sepsis is based on the latest literature (13). Clinical data and blood samples were collected upon patient admission. The blood samples were centrifuged at 4°C (1,000 × g for 15 min) within 30 min of collection to obtain serum, which was subsequently stored at −80°C for further analysis. This study received approval from the Ethics Committee of the Hunan Children's Hospital (approval number: KYSQ2021-194). Prior to participation, each patient's guardian provided informed consent by signing a consent form.

### 2.2 Animals and groups

Twenty-four C57BL/6 male mice aged 8–10 weeks and weighing 22–26 g were purchased from experimental animal center of Xiangya Medical College (Changsha, Hunan, China). All the experiments were performed in accordance with the ARRIVE guidelines for the Care and Use of Laboratory Animals and Ethics Committee of Hunan Children's Hospital. Prior to the experiment, the mice were acclimatized to a controlled indoor environment for over 1 week without any restrictions on food or water intake. This was followed by cecal ligation and puncture (CLP) to induce sepsis-associated pancreatic injury. The mice were randomly divided into three groups (*n* = 8): SPI group, SPI + PSP/reg 40 ng/kg group, SPI+PSP/reg 400 ng/kg group. After CLP surgery, mice in SPI+PSP/reg 40 ng/kg group and SPI+PSP/reg 400 ng/kg group were intraperitoneally injected with 40 ng/kg PSP/reg (Sino Biological, dissolved in 100 μl PBS) and 400 ng/kg PSP/reg daily. Mice in SPI group were received normal saline at a same dose. After 3 days of intervention, all mice were euthanized via inhalation of 3% isoflurane. Blood samples and pancreatic tissues were subsequently collected and separated.

### 2.3 Isolation and culture of primary pancreatic acinar cells

The primary pancreatic acinar cells (PACs) were isolated from C57BL/6 mice using a collagenase digestion method as previously described (14). Fresh pancreatic tissues were carefully dissected and rinsed three times with phosphate-buffered saline. Subsequently, 200 U/ml of collagenase IV was gently infused into the pancreas. The pancreatic sample was then subjected to a water bath at 37°C for the digestion period. After digestion, the pancreatic sample was immersed in an extracellular solution meticulously calibrated to maintain a pH of 7.3. To ensure thorough homogenization, the digested sample underwent repetitive pipetting within this solution. The resultant suspension was then filtered through a sterile cell strainer to remove any particulate matter. Following this step, gentle centrifugation at 700 rpm for 2 min was performed. Subsequently, the cells were cultured in DMEM supplemented with 10% fetal bovine serum and incubated at a controlled temperature of 37°C.

### 2.4 Serum amylase measurement

The assessment of pancreatic injury was conducted with meticulous attention to detail through the analysis of serum amylase activity. The measurement of serum amylase levels was carried out using a specialized assay kit (Sigma-Aldrich), in strict adherence to the protocols established by the manufacturer.

### 2.5 PACs culture and treatment

Primary pancreatic acinar cells (PACs) were cultured in Ham's F12 medium and subsequently seeded into 6-well cluster dishes at a density of 5 × 10^5^ cells/ml. The cultures were then incubated in a humidified chamber maintained at 37°C with an atmosphere consisting of 5% CO_2_ and 95% ambient air. This study on PACs comprises three experimental groups as follows: (1) PACs +LPS (10 μg/ml) group (control group), (2) PACs + LPS (10 μg/ml) +PSP/reg (100 nM) group, (3) PACs+LPS (10 μg/ml) +PSP/reg (1,000 nM) group. The PACs in each group were cultured for a duration of 24 h.

### 2.6 Hematoxylin and eosin staining analysis

Fresh pancreatic tissues were fixed in formalin for 24 h. Following fixation, the tissues were embedded in paraffin and sectioned to a thickness of approximately 5 μm for each group. The sections were stained using a hematoxylin and eosin (H&E) staining kit according to the manufacturer's protocol. Subsequently, the histopathological injury scores of the pancreas were evaluated under light microscopy by two pathologists who performed their assessments blindly, based on previously established criteria (15).

### 2.7 Hoechst 33342/PI staining

Hoechst 33342/PI staining was employed to detect pyroptosis in pancreatic acinar cells. The cells were carefully plated in six-well plates and allowed to incubate for a period of 24 h. Following this incubation, the cells underwent distinct treatments. Subsequently, they were washed twice with pre-cooled PBS. To facilitate cellular visualization, a staining mixture consisting of Hoechst 33342 and PI (1 ml staining buffer + 5 μl Hoechst 33342 + 5 μl PI) was applied to the cells and incubated in the dark at 4°C for exactly 20 min. The stained cells were then promptly observed using an inverted fluorescence microscope (Olympus, Japan). Quantification of PI-stained cells was performed utilizing Image J software for comprehensive analysis.

### 2.8 Immunohistochemical analysis

Initially, 4% formalin was utilized to meticulously fix pancreatic tissues prior to their embedding in paraffin. The paraffin-embedded pancreatic tissues were subsequently sectioned into ~5 μm slices for the purpose of immunohistochemistry. In brief, the paraffin sections underwent a dewaxing process, followed by soaking and washing, before being incubated with 3% H_2_O_2_ at room temperature for 20 min to inhibit endogenous peroxidase activity. The samples were then subjected to an overnight incubation with the primary antibody (caspase-1, dilution 1:200, PA5-99390; Thermo Fisher Scientific) at a temperature of 4°C. Following this step, the sections received treatment with corresponding secondary antibodies. Furthermore, color development was achieved using 3, 3′-diaminobenzidine (DAB), followed by hematoxylin staining. Subsequently, alcohol dehydration and cleaning procedures were performed before gumming the slides. The prepared samples were visualized under a light microscope (Olympus AG, Zurich, Switzerland).

### 2.9 Immunofluorescence staining

Briefly, pancreatic acinar cells were meticulously cultured in 6-well plates at a density of 5 × 10^5^ cells per well. Following a culture and treatment period of 24 h, the cells were incubated with anti-caspase-1 (dilution: 1:100, PA5-99390, Thermo Fisher Scientific) overnight at a temperature of 4°C. After three thorough washes with PBS, the cells were subsequently incubated with the appropriate secondary antibody at room temperature. To visualize cellular nuclei, counterstaining was performed using DAPI at a concentration of 1 μg/ml under ambient conditions. Fluorescent images were then captured utilizing an Olympus fluorescence microscope (Tokyo, Japan) at a magnification level of 400 ×.

### 2.10 Western blot analysis

Proteins were meticulously extracted from pancreatic tissues and pancreatic acinar cells by utilizing a protein extraction buffer kit in strict accordance with the manufacturer's detailed protocol. The BCA protein assay kit was employed to accurately quantify the protein content. Subsequent Western blot analyses were performed following established methodologies, using primary antibodies specific for NLRP3 (ab263899, 1:500, Abcam), cleaved caspase-1 (PA5-99390, 1:500, Thermo Fisher Scientific), and GSDMD-N (36425S, 1:500, Cell Signaling Technology). For the detection of chemiluminescent signals, an enhanced chemiluminescence substrate kit (WBKLS0100, Merck Millipore) was utilized. The membranes were imaged and quantified through densitometry using the ChemiDoc XRS+ system (Bio-Rad Co., USA), facilitating precise analysis of the experimental results.

### 2.11 Enzyme-linked immunosorbent assay

The levels of PSP/reg (Fine Biotech, China), TNF-α, IL-6, IL-1β and IL-18 (BOSTER, China), as well as LDH (Cloud-Clone Corp USCN Life Science, China), in serum were assessed using commercial ELISA kits in accordance with the manufacturer's protocols.

### 2.12 Statistical analysis

The statistical analysis was performed using SPSS version 26.0 and GraphPad Prism version 8.0, with results expressed as the mean ± standard deviation or median (interquartile range [IQR]). Statistical analyses included ANOVA for continuous variables and chi-square tests for categorical data, with Bonferroni corrections for multiple comparisons. The Spearman rank correlation test was performed to analyze the correlation between circulating PSP/Reg levels and disease severity or levels of pancreatic amylase. Logistic regression and receiver operating characteristic (ROC) analyses were performed to assess whether PSP/Reg was a predictive factor for mortality. Test results were reported as two-tailed *P* values, with a *P* < 0.05 considered statistically significant.

## 3 Results

### 3.1 Increased circulating PSP/reg levels in children with sepsis admitted to the PICU

To assess the clinical significance of circulating PSP/reg levels, 137 children diagnosed with sepsis and admitted to the PICU were enrolled in this study. There were 91 male cases (66.4%) and 46 female cases (33.6%). Among them, 73 cases (53.3%) were under 1 year old, 33 cases (24.1%) were between 1 and 3 years old, and 31 cases (22.6%) were over 3 years old. Respiratory system infections accounted for 81 cases (59.1%), nervous system infections for 32 cases (23.3%), digestive system infections for 16 cases (11.6%), and other infections for 8 cases (6.0%). Among the included patients, 101 (74%) survived (survival group) and 36 (26%) died (dead group). Among the 137 critically ill children, they were classified into the normal pancreatic amylase group (94, 68.6%), the mild elevation of pancreatic amylase group (Increase by 1 to 3 times) (25, 18.2%) and the severe elevation of pancreatic amylase group (Increased by more than three times) (18, 13.2%) based on the levels of pancreatic amylase; and they were also divided into the sepsis group (75, 54.7%), the severe sepsis group (41, 29.9%) and the septic shock group (21, 15.4%) according to the disease severity.

**Figure 1:**
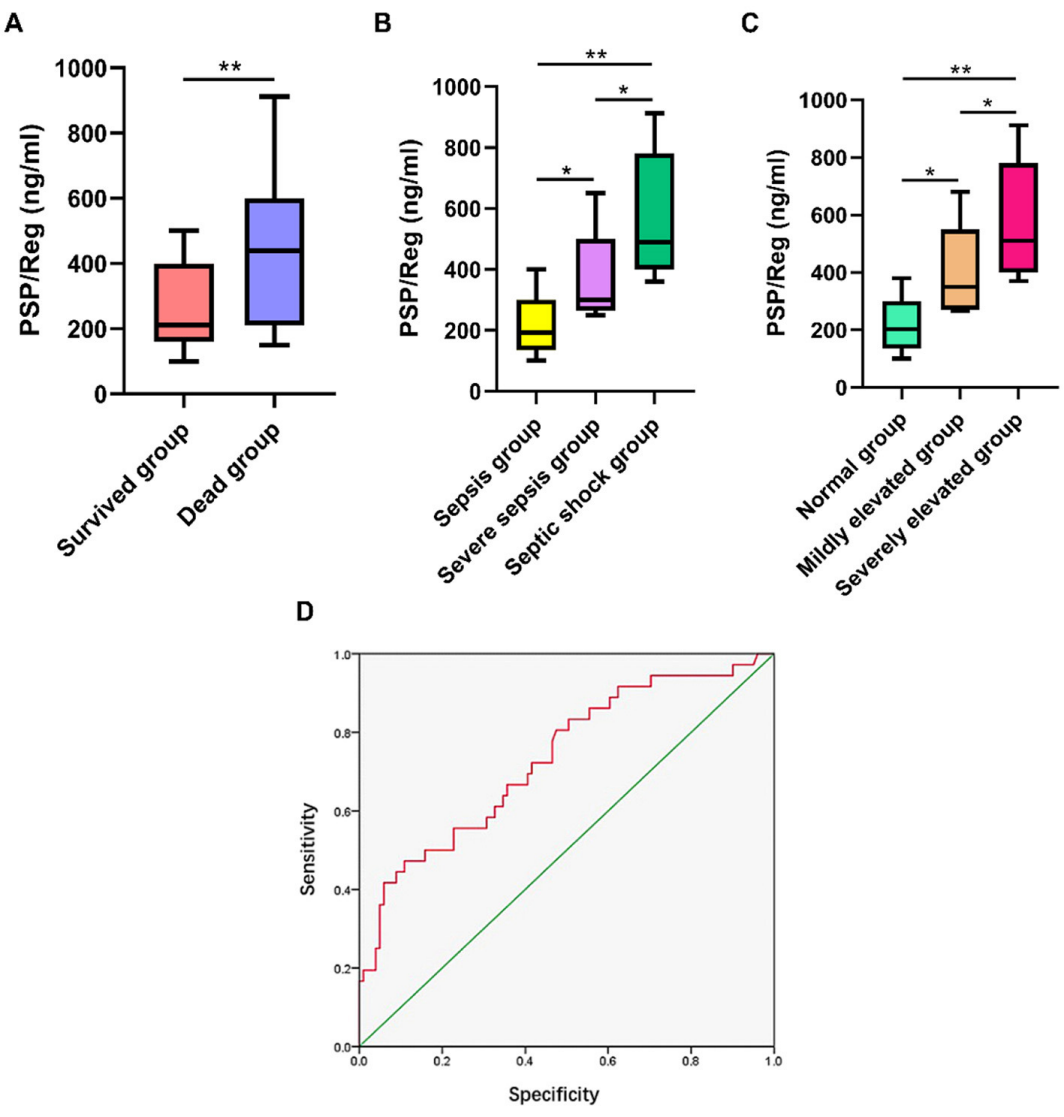
Increased circulating PSP/reg levels in children with sepsis admitted to the PICU. **(A)** Circulating PSP/reg levels were compared between survived group and dead group. **(B)** Circulating PSP/reg levels were compared according to disease severity in children. **(C)** Circulating PSP/reg levels were compared according to pancreatic amylase in children. **(D)** The receiver operating characteristic curve analysis of PSP/Reg for the prediction of mortality. *P < 0.05, **P < 0.01.

Children in dead group exhibited higher circulating PSP/reg levels compared with children in survival group (median, 601.8 ng/ml [IQR, 507.1–687.5 ng/ml] vs. 278.8 ng/ml [IQR, 178.9–341.7 ng/ml]) (*P* < 0.01) (Figure 1A). The pediatric clinical illness score (PCIS) of the death group (71.06 ± 8.92) was significantly lower than that of the survival group (82.23 ± 6.40) (*P* < 0.01). The circulating PSP/reg level in the septic shock group (median, 611.1 ng/ml [IQR, 477.8–795.2 ng/ml]) was significantly higher than that in the sepsis group (median, 209.9 ng/ml [IQR, 165.2–307.7 ng/ml]) (*P* < 0.01) and the severe sepsis group (median, 409.3 ng/ml [IQR, 327.3–557.1 ng/ml]) (*P* < 0.05) (Figure 1B). Similarly, the circulating PSP/reg level in the severe elevation of pancreatic amylase group (median, 683.8 ng/ml [IQR, 539.6–810.2 ng/ml]) was significantly higher than that in the normal pancreatic amylase group (median, 270.2 ng/ml [IQR, 170.8–330.1 ng/ml]) (*P* < 0.01) and the mild elevation of pancreatic amylase group (median, 501.2 ng/ml [IQR, 399.8–616.3 ng/ml]) (*P* < 0.05) (Figure 1C). The ROC analysis also revealed that PSP/reg was a predictor of mortality (area under the ROC curve, 0.733, 95%CI: 0.635–0.831, *P* < 0.001) (Figure 1D). The Spearman rank correlation test demonstrated a strong correlation between circulating PSP/reg levels and disease severity (*r*=0.503, *P* < 0.001) and levels of pancreatic amylase (*r*=0.571, *P* < 0.001). After adjusting for age and sex, the log regression model showed that the PSP/Reg level, procalcitonin (PCT), PCIS score ≤ 70, number of MODS ≥ 3 and shock were risk factor for progression to mortality (Table 1).

**Table 1:** Multivariate logistic regression analysis of risk factors for the prognosis in children.

**Variables**	**β**	**SE**	**Wald χ^2^**	***P***	**OR (95%CI)**
PSP/Reg	1.239	0.475	7.021	0.007	3.421 (1.378–8.594)
PCT	0.510	0.161	10.396	0.001	1.652 (1.226–2.234)
PCIS ≤ 70	0.042	0.009	22.375	0.000	1.043 (1.023–1.070)
MODS ≥ 3	0.487	0.068	47.091	0.000	1.613 (1.401–1.854)
Shock	1.168	0.459	6.519	0.011	3.229 (1.301–7.916)

### 3.2 Exogenous PSP/reg attenuated the severity of sepsis-associated pancreatic injury

**Figure 2:**
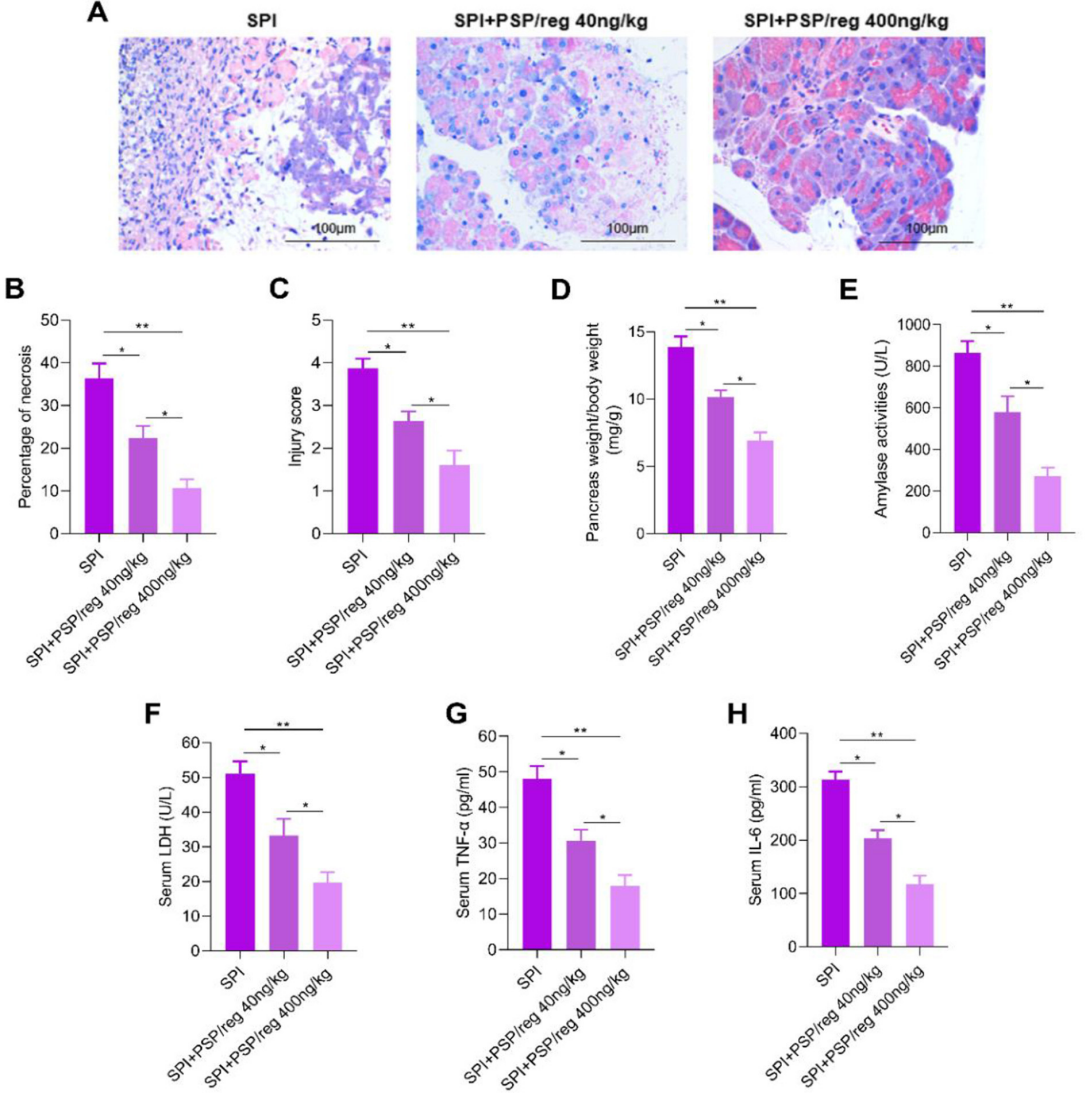
PSP/reg attenuated the severity of sepsis-associated pancreatic injury. **(A)** H&E staining of mouse pancreatic tissues. **(B)** Percentages of necrotic areas. **(C)** Histological scores of pancreatic tissues. **(D)** Pancreas weight relative to body weight. **(E)** Serum amylase levels. **(F)** Serum LDH levels were determined. **(G)** Serum TNF-α levels. **(H)** Serum IL-6 levels. Values were means ± SD, *n* = no. of animals. *P < 0.05, **P < 0.01.

The effects of PSP/reg on CLP-induced sepsis-associated pancreatic injury were evaluated. As shown in Figure 2A, CLP induced pancreatic injury, characterized by inflammation, edema, hyperemia, vacuolization and necrosis, while PSP/reg administration mitigated pancreatic tissue injury. Compared to the SPI group, pancreatic pathological injury was found to be attenuated in the SPI + PSP/reg group. PSP/reg administration markedly decreased necrosis area (Figure 2B) and injury scores (Figure 2C). Besides, we determined the amylase activity and pancreas weight/body weight to assess the degree of pancreatic injury. The results showed that the amylase activity and pancreas weight/body weight decreased significantly in PSP/reg-treated mice compared to the SPI mice (Figures 2D, E). Additionally, the serum levels of LDH, TNF-α and IL-6 were also remarkably downregulated in PSP/reg-treated mice compared to the SPI mice (Figures 2F–H). These results suggested that exogenous PSP/reg attenuated the severity of sepsis-associated pancreatic injury.

### 3.3 Exogenous PSP/reg inhibited pancreatic pyroptosis in sepsis-associated pancreatic injury model

**Figure 3:**
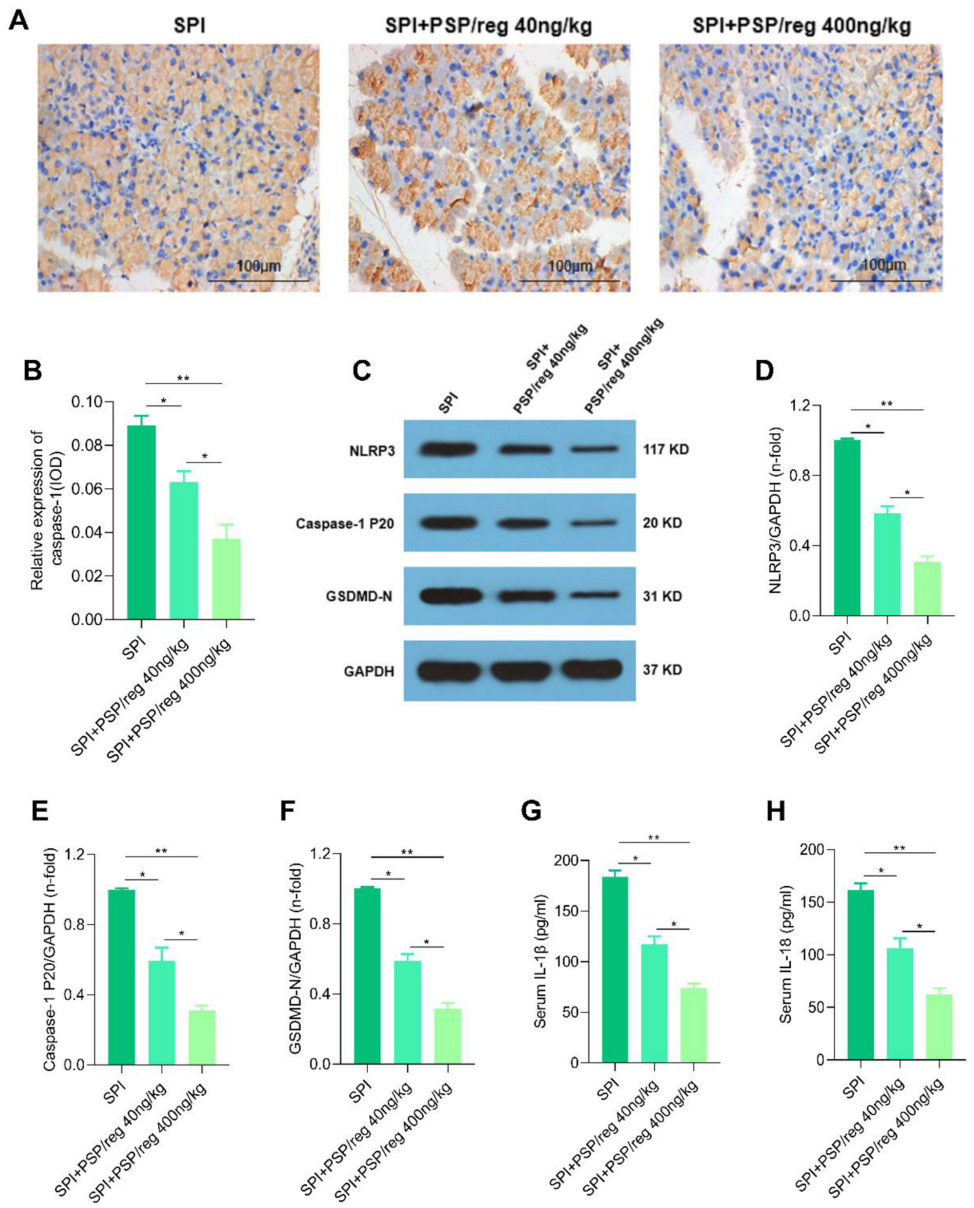
PSP/reg inhibited pancreatic pyroptosis in sepsis-associated pancreatic injury model. **(A)** Representative IHC images of the Caspase-1 expression in the pancreatic tissues. **(B)** Relative expression level of caspase-1 was analyzed by histogram (*n* = 8). **(C)** Protein levels of NLRP3, Caspase-1 p20 and GSDMD-N in the pancreatic tissues analyzed by Western blot. **(D–F)** Quantitative analysis of NLRP3, Caspase-1 p20 and GSDMD-N in the pancreatic tissues (*n* = 5). **(G)** Serum IL-1β levels (*n* = 8). **(H)** Serum IL-18 levels (*n* = 8). Values were means ± SD. *P < 0.05, **P < 0.01.

A subsequent study was designed to explore whether PSP/reg inhibited the pyroptosis *in vivo*. First, IHC staining of the lungs showed that the expression of caspase-1 was remarkably downregulated in PSP/reg-treated mice compared to the SPI mice (Figures 3A, B). Western blot confirmed that the expressions of pyroptosis activation-associated proteins, NLRP3, Caspase-1 p20 and GSDMD-N in the pancreatic tissues were significantly lesser in PSP/reg-treated mice compared to the SPI mice (Figures 3C–F). Next, we found the levels of inflammatory cytokines IL-1β and IL-18 of serum were all decreased in PSP/reg-treated mice compared to the SPI mice (Figures 3G, H). These results suggested that exogenous PSP/reg inhibited pancreatic pyroptosis in sepsis-associated pancreatic injury model.

### 3.4 Exogenous inhibited pyroptosis in LPS-stimulated pancreatic acinar cells

**Figure 4:**
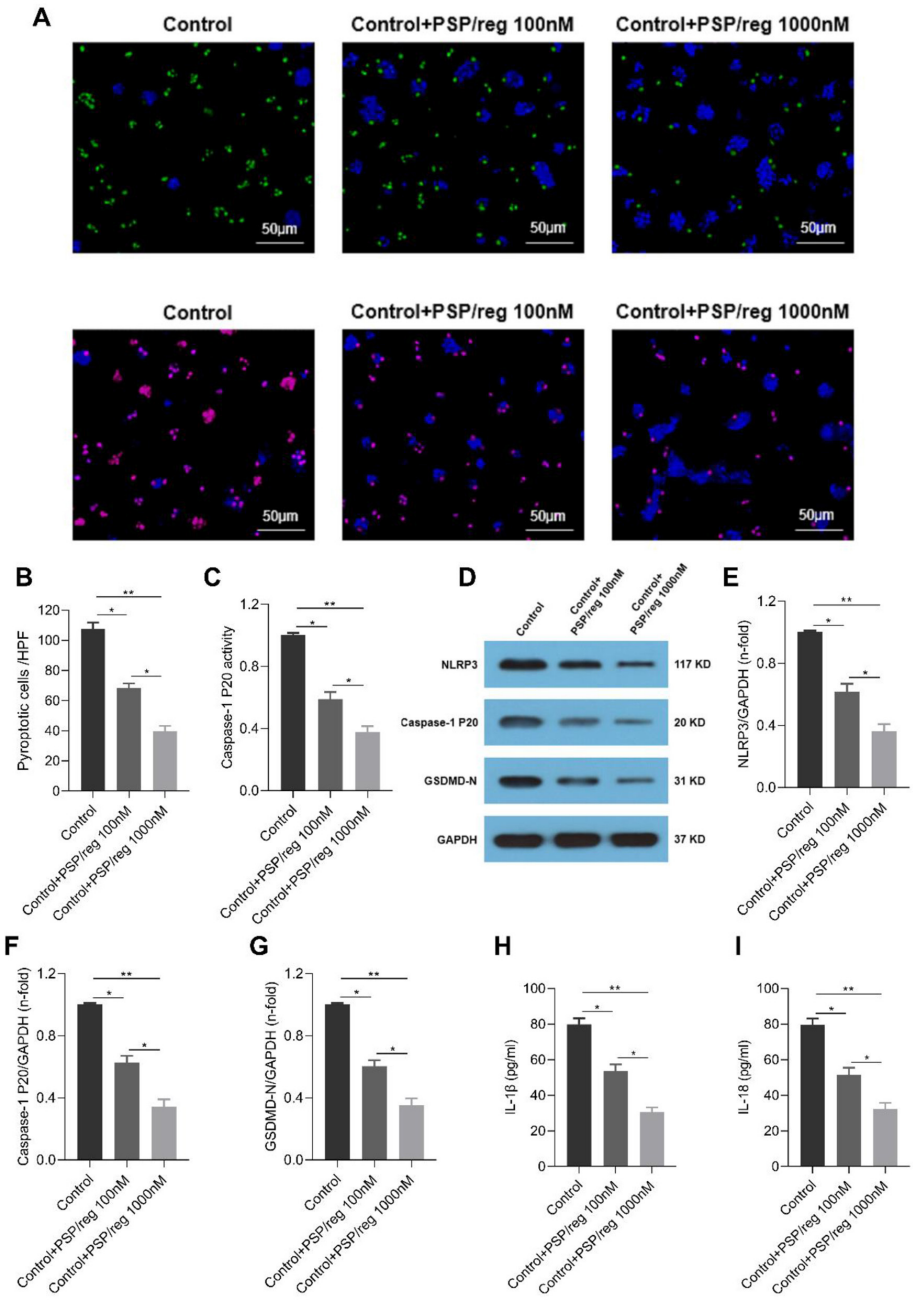
PSP/reg inhibited pyroptosis in LPS-stimulated pancreatic acinar cells. **(A)** Double immunofluorescence staining of caspase-1 in pancreatic acinar cells. Pyroptosis in pancreatic acinar cells was examined by Hoechst33342/PI staining. **(B)** Proportion of pyroptotic cells were quantified by histogram (*n* = 6). **(C)** Analysis of the caspase-1 activity in pancreatic acinar cells (*n* = 6). **(D)** Western blot analysis was used to determine the protein levels of NLRP3, Caspase-1 p20 and GSDMD-N in pancreatic acinar cells. **(E–G)** Quantitative analysis of the relative expression levels of NLRP3, Caspase-1 p20 and GSDMD-N (*n* = 5). **(H, I)** The levels of 1β and IL-18 in the culture supernatant of pancreatic acinar cells were determined by ELISA (*n* = 6). Values were means ±SD. *P < 0.05, **P < 0.01.

We further investigated whether PSP/reg could inhibit pyroptosis in LPS-stimulated pancreatic acinar cells *in vitro*. Immunofluorescent staining showed that caspase-1 activity was markably decreased in PSP/reg-treated group compared to the control group. Besides, Hoechst/PI staining revealed that PSP/reg administration significantly alleviated the LPS-induced pyroptosis (Figures 4A–C). In addition, Western blot analysis showed that pyroptosis activation-associated proteins, NLRP3 (Figure 3H), Caspase-1 p20 (Figure 3I) and GSDMD-N (Figure 3J) in pancreatic acinar cells were greatly elevated following LPS stimulation, which decreased with PSP/reg treatment (Figures 4D–G). The ELISA results showed that the levels of IL-1β and IL-18 in the supernatant of PSP/reg-treated group were significantly lower than those in the control group (Figures 4H, I). Taken together, consistent with findings *in vivo*, these results indicated that PSP/reg inhibited pyroptosis in LPS-stimulated pancreatic acinar cells.

## 4 Discussion

Sepsis is a life-threatening condition characterized by an dysregulated host response to infection, which ultimately leads to organ dysfunction (16). Despite significant advances in our understanding of its pathology, sepsis continues to pose a global health challenge and remains a major contributor to mortality rates. Timely identification of this condition is crucial for improving clinical outcomes, as delays in treatment can have a profound impact on survival. Consequently, biomarkers play an essential role in the diagnosis, risk stratification, and management of sepsis (17). Pancreatic stone protein (PSP), a C-type lectin, has recently been investigated as a potential biomarker for sepsis. The existing evidence indicates that PSP demonstrates superior diagnostic performance in identifying infections compared to the most commonly used biomarkers, and it also provides additional prognostic value (18). Increasing evidence suggested that serum PSP/reg is a biomarker related to organ failure and outcome in patients with ventilator-associated pneumonia (19). PSP/reg could predict unfavorable outcomes, such as sepsis development, readmission, and the need for treatment escalation among patients with intra-abdominal infections (20). In this current research, children in dead group exhibited higher circulating PSP/reg levels compared with children in survival group. The circulating PSP/reg level in the septic shock group was significantly higher than that in the sepsis group and the severe sepsis group. Thus, these data may indicate PSP/reg exhibited higher sensitivity and specificity in the diagnosis of sepsis. It facilitated the early detection of sepsis and allows for a certain degree of assessment regarding the severity of septic patients.

Our previous clinical study demonstrated that serum amylase levels may serve as an independent biomarker for predicting pancreatic injury in critically ill children (4). A recent study by Hu and colleagues demonstrated that circulating PSP/Reg levels were correlated with the prognosis and sequential organ failure assessment scores (21). In this study, the circulating PSP/reg level in the severe elevation of pancreatic amylase group was significantly higher than that in the normal pancreatic amylase group and the mild elevation of pancreatic amylase group. Circulating PSP/reg levels were correlated with pancreatic amylase. Thus, our study demonstrated that PSP/reg levels could reflect the degree of pancreatic injury to some extent.

The pancreas responds to distant lesions and septic insults in mice and rats by increasing the synthesis of PSP/Reg. Moreover, in septic patients, serum PSP/Reg is predominantly derived from an acute phase response initiated by the pancreas (22). Another study indicated that administration of anti-PSP/Reg antibodies exacerbated acute pancreatitis, suggesting that the PSP/Reg played a protective role in this condition (23). Our data revealed that PSP/reg administration mitigated pancreatic tissue injury and decreased injury scores and necrosis area. Besides, the amylase activity, the serum levels of LDH, TNF-α and IL-6 were remarkably downregulated in PSP/reg-treated mice compared to the SPI mice. These findings indicated that PSP/reg attenuated the pancreatic injury and exerted a protective effect in sepsis-associated pancreatic injury.

The pathophysiology of pancreatic injury in patients suffering from sepsis or septic shock remains incompletely elucidated. The prevailing hypothesis attributes this injury primarily to pancreatic ischemia. However, a limited number of studies suggested that additional mechanisms may also contribute, including cellular apoptosis, increased nitric oxide release from endothelial cells, platelet activation, the ischemia-reperfusion phenomenon, elevated triglyceride levels, and the formation of biliary sludge (24). Understanding the molecular mechanisms underlying pancreatic injury and identifying its associated pathways are essential for the development of effective pharmacological therapies aimed at preventing the progression of sepsis-associated pancreatic injury. Numerous studies have reported pyroptosis was a critical factor in the pathogenesis and progression of sepsis, septic shock, and organ dysfunction (25). For example, Li et al. found that promotion of activating transcription factor 3 (ATF3) could attenuate sepsis-induced acute lung injury (ALI) by regulating pyroptotic pathways (26). Additionally, Helix B surface peptide (HBSP) protected LPS-induced acute kidney injury by inhibiting tubular cell pyroptosis (27). Inhibition of NLRP3 inflammasome-induced pyroptosis could alleviate sepsis-associated intestinal injury (28). Our data also revealed that the expressions of pyroptosis activation-associated proteins, NLRP3, caspase-1 p20 and GSDMD-N in the pancreatic tissues were significantly greater in the SPI group. These findings indicated that the occurrence of SPI was accompanied by induction of pyroptosis, targeting of pyroptosis might be a potential strategy for the treatment of sepsis-associated pancreatic injury.

It has been established through a series of studies that pyroptosis, which is mediated by the NLRP3 inflammasome, plays a crucial role in the pathophysiology of sepsis (29). Activated caspase-1 plays a crucial role in cleaving the GSDMD protein molecule, thereby initiating the oligomerization of its amino terminus (GSDMD-N). This process facilitates the formation of pores in the plasma membrane and promotes the secretion of IL-1β and IL-18. Ultimately, this cascade leads to pyroptosis, which is implicated in the pathogenesis of systemic inflammation (30). Recent studies have shown the key role of NLRP3/caspase-1/GSDMD-dependent pyroptosis signaling pathway in the regulation of sepsis-associated organ dysfunction. For instance, phillyrin prevented sepsis-induced acute lung injury through inhibiting the NLRP3/caspase-1/GSDMD-dependent pyroptosis signaling pathway (31). Similarly, puerarin protected against sepsis-associated encephalopathy by inhibiting NLRP3/Caspase-1/GSDMD pyroptosis pathway and reducing blood-brain barrier damage (32). In addition, phospholipase D2 (PLD2) deletion ameliorates sepsis-induced cardiomyopathy by suppressing cardiomyocyte pyroptosis via the NLRP3/caspase-1/GSDMD pathway (33). Our data demonstrated that pyroptosis activation-associated proteins, NLRP3, Caspase-1 p20 and GSDMD-N in pancreatic acinar cells were greatly elevated following LPS stimulation. These results elucidated the important role of NLRP3/caspase-1/GSDMD pathway in sepsis-associated pancreatic injury.

Previous study indicated that PSP/reg might have a protective function in the repair phase of acute and chronic pancreatitis by promoting resolution of fibrosis (34). More importantly, PSP/reg exhibited the anti-apoptotic effect in many other diseases (35–37). In this study, our results showed the expressions of pyroptosis activation-associated proteins, NLRP3, Caspase-1 p20 and GSDMD-N in the pancreatic tissues and pancreatic acinar cells were significantly lesser in PSP/reg-treated group compared to the SPI or control group. Additionally, the levels of inflammatory cytokines IL-1β and IL-18 were all decreased in PSP/reg-treated group compared to the SPI or control group. These results indicated that PSP/reg inhibited pyroptosis *in vivo* and *in vitro*. To our knowledge, these findings indicated for the first time that PSP/reg confers protection against sepsis-associated pancreatic injury through the inhibition of NLRP3/caspase-1/GSDMD-dependent pyroptosis signaling pathway activation. In published data, Purified PSP/reg can itself act as an autocrine factor for targeting a pathway involving NF-kappaB (NF-κB) (38). NF-κB is an important upstream regulatory factor of NLRP3/caspase-1/GSDMD pathway (39, 40). Thus, it was speculated that PSP/reg regulated NLRP3/caspase-1/GSDMD pathway by inhibiting NF-κB.

Several limitations should be mentioned for this study. First, although PSP does not cause harm to pancreatic tissue, we still need to include a “PSP/reg-only” treatment group to confirm that PSP/reg alone does not induce any harmful effects on pancreatic tissue. Moreover, we only conducted the phenotypic studies, the specific mechanism of inhibiting apoptosis of pancreatic cells will be further explored in subsequent studies. Second, previous study has demonstrated that the serum PSP level gradually increased within 72 h during the course of sepsis (41). Thus, we should investigate the PSP levels of patients with sepsis at different time points, such as 48 h, 72 h, and even the 7th day, to observe the changes of PSP during the course of the disease. Third, our previous multicenter study has shown that renal insufficiency was not the risk factor for elevated amylase (3). Nevertheless, renal function definitely has an impact on the changes of amylase to some extent. In the subsequent studies, we should exclude patients with renal insufficiency, and collect the data of abdominal CT or B-ultrasound of patients with sepsis to explore the diagnostic value of PSP combined with abdominal CT or B-ultrasound for sepsis-associated pancreatic injury.

In summary, we undertook this research that increased circulating PSP/reg levels were observed in sepsis-associated pancreatic injury patients. PSP/reg may exhibit protective effects by inhibiting pancreatic pyroptosis in sepsis-associated pancreatic injury model and LPS-stimulated pancreatic acinar cells. Our study also provided new clues to understand the function of PSP/reg and raised the possibility of promising target for the treatment of sepsis-associated pancreatic injury.

## Data Availability

The original contributions presented in the study are included in the article/supplementary material, further inquiries can be directed to the corresponding author.
